# Acute Coronary Syndrome (ACS) due to Coronary Artery Embolism in a Patient with Atrial Fibrillation

**DOI:** 10.1155/2019/9347198

**Published:** 2019-10-10

**Authors:** Hussein Daoud, Ashraf Abugroun, Shruti Erramilli, Surender Kumar

**Affiliations:** ^1^Department of Internal Medicine, Advocate Illinois Masonic Medical Center, 836 W Wellington Ave., Chicago, IL 60657, USA; ^2^Department of Cardiology, Advocate Illinois Masonic Medical Center, 836 W Wellington Ave., Chicago, IL 60657, USA

## Abstract

Acute coronary syndrome (ACS) secondary to a coronary embolism is an unusual occurrence, yet an important consideration given the difficult diagnosis. We report a case of a 69-year-old male with a medical history of paroxysmal atrial fibrillation who presented with chest pain and shortness of breath. A coronary angiogram was significant for three focal transluminal and translucent areas in the ostial, mid, and distal circumflex artery consistent with embolic disease. The patient was subsequently managed medically with anticoagulation. Despite being a relatively rare entity, thromboembolism into the coronary arteries can provoke an acute myocardial infarction, with atrial fibrillation being the most common risk factor. Treatment modalities for ACS secondary to thromboembolism include stent placement, intracoronary thrombolysis, and thrombus aspiration.

## 1. Introduction

Acute myocardial infarction secondary to a coronary embolism was first reported in 1856 by the famous German physician Rudolf Virchow [1]. Approximately 3% of acute coronary syndromes (ACS) are caused by a coronary embolism. Coronary emboli can arise through various mechanisms including a left atrial appendage thrombus due to atrial fibrillation, valve vegetations secondary to infective or autoimmune endocarditis, deep venous thrombi embolizing paradoxically through a patent foramen ovale (PFO), or iatrogenic secondary to coronary interventions [2]. Of these, atrial fibrillation is thought to be the most common etiology of coronary emboli, though there are few cases reported in the literature. Particularly, a non-ST-segment elevation myocardial infarction (NSTEMI) caused by embolic occlusion of a coronary artery in the setting of atrial fibrillation is a rare entity. The clinical presentation is like ACS of any cause. In this report, we discuss a case of a 69-year-old male with a history of paroxysmal atrial fibrillation who presented with chest pain and exertional dyspnea. The coronary angiogram demonstrated three lesions in the ostial, mid, and distal circumflex artery consistent with emboli.

## 2. Case Report

A 69-year-old male presented to the emergency department complaining of chest pain and shortness of breath. His medical history was significant for paroxysmal atrial fibrillation on warfarin, coronary artery disease (CAD) with a proximal left anterior descending artery (LAD) stent, balloon valvuloplasty for severe aortic stenosis, and end-stage renal disease (ESRD) treated with peritoneal dialysis. The patient reported intermittent chest tightness that worsened with exertion and that was associated with progressive shortness of breath for one week before hospital arrival.

**Figure 1:**
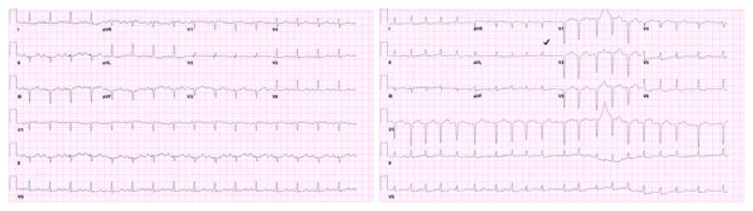
Admission electrocardiogram (ECG) on the left with a normal sinus rhythm and ECG on the right taken during the hospital stay showing atrial fibrillation with a rapid ventricular response.

On admission, his heart rate was 104 beats per minute, blood pressure was 104/67 mmHg, respiratory rate was 22 breaths per minute, and the patient's oxygen saturation was above 94% on ambient air. The physical exam revealed a grade II/VI systolic ejection murmur over the aortic valve area. Labs showed a potassium of 5.8, BUN of 65 mg/dL, creatinine of 12.47 mg/dL (unknown baseline), hemoglobin of 8.4 g/dL, and a troponin I of 2.76 ng/dL (normal < 0.05) that peaked on repeat at 3.79 ng/dL. His international normalized ratio (INR) on admission was 2.0, though his compliance with anticoagulation and the INR in the weeks leading up to his admission was unknown. A chest X-ray was remarkable for mild cardiomegaly, interstitial pulmonary edema, and trace bilateral pleural effusions. An electrocardiogram (ECG) showed a normal sinus rhythm with an old apical infarct, and a transthoracic echocardiogram (TTE) revealed an ejection fraction of 15-20% with grade two diastolic dysfunction and “possible” severe aortic valve stenosis (Figure 1).

**Figure 2:**
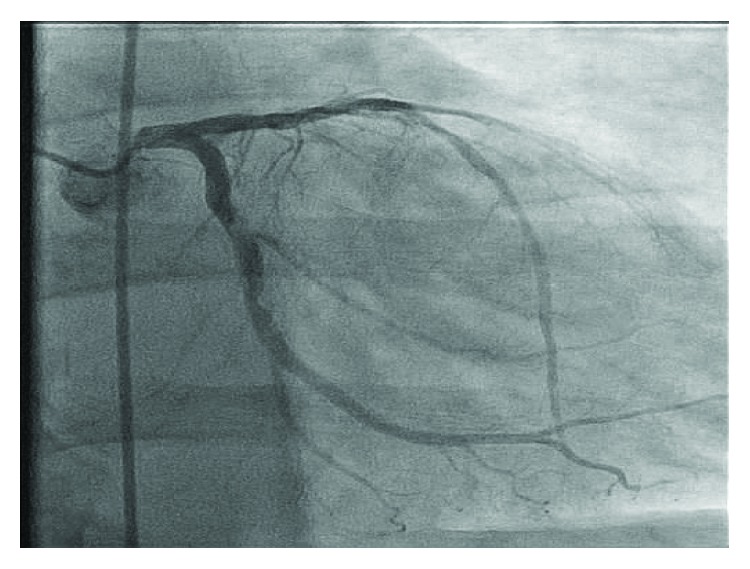
Left heart catheterization showing three focal transluminal and translucent areas in the ostial, mid, and distal circumflex artery that was mobile and hazy appearing—concerning for emboli.

The patient was diagnosed with a non-ST-segment elevation myocardial infarction (NSTEMI) and underwent both a right- and left-sided heart catheterization. The coronary angiogram revealed three focal transluminal and translucent areas in the ostial, mid, and distal circumflex artery consistent with coronary emboli (Figure 2). The left circumflex artery was otherwise angiographically normal. Given the patient's low ejection fraction and history of severe aortic stenosis, the interventional cardiologist recommended medical management with anticoagulation, instead of potential thrombolysis or thrombus aspiration, as he would be high risk for any coronary intervention.

A transesophageal echocardiogram (TEE) with agitated saline was subsequently ordered and did not reveal a patent foramen ovale (PFO), atrial septal defect (ASD), evidence of an embolic source (in the left atrial appendage or left ventricle), or any valve vegetation. The patient denied any episodes of chest pain or dyspnea during his hospital stay. He was deemed a poor candidate for aortic valve surgery by cardiothoracic surgery and was ultimately discharged on his home doses of aspirin, carvedilol, amiodarone, and warfarin with a follow-up for continued evaluation for a transcatheter aortic valve replacement (TAVR) procedure as his prior balloon valvuloplasty was completed as a bridging intervention.

## 3. Discussion

Individuals with atrial fibrillation are at an increased risk of thrombus formation in the left atrium, particularly in the left atrial appendage, through Virchow's triad of blood stasis, endothelial damage, and hypercoagulable state. They may also develop nonleft atrial appendage thrombi due to the overall diseased state of the atrium (atriopathy). This subsequently puts this population at an increased risk of thromboembolic events of the systemic circulation. Thromboembolism most often occurs during episodes of fibrillation or within the first ten days following cardioversion back to sinus rhythm. Sites of systemic embolism include the brain, splenic artery, renal artery, mesenteric artery, and limbs [3].

Myocardial infarction has been known to cause atrial fibrillation through multiple mechanisms, including ventricular ischemia/infarction and subsequent atrial stretching or directly through atrial ischemia [4]. Conversely, atrial fibrillation has been linked to myocardial infarction through its association with multiple atherosclerotic risk factors including diabetes, hypertension, and dyslipidemia. Additionally, atrial fibrillation is associated with inflammation that may promote a prothrombotic state and subsequent myocardial infarction. When these two conditions coincide, clinical management becomes more complex given the higher risk of bleeding when combining anticoagulant and antiplatelet medications [5].

The phenomenon of thromboembolism into the coronary arteries is rare with an unknown prevalence given its difficult diagnosis. Acute myocardial infarction secondary to a coronary embolism was first reported in 1856 by the famous German physician Rudolf Virchow [1]. In patients with myocardial infarction, 4-7% are found to not have atherosclerotic vascular disease [6]. In an autopsy study of 419 patients, 55 (13%) were found to have an embolic infarct in their coronary arteries [7]. A study by Shibata et al. looked at new-onset acute myocardial infarctions between 2001 and 2013, and 1776 consecutive cases were analyzed with a focus on coronary embolism (CE) diagnosis through angiography, histology, and imaging. In that study, 52 patients were acknowledged to have CE (2.9%) and the most common etiologies were atrial fibrillation, cardiomyopathy, and valvular heart disease with a prevalence of 73%, 25%, and 15%, respectively [8].

Therefore, even though atrial fibrillation is thought to be the most common risk factor, other potential sources for coronary embolization exist. These include a left ventricular mural thrombus from a prior myocardial infarction, thromboemboli from the trabeculated myocardium in noncompaction cardiomyopathy, or intrapulmonary shunting through arteriovenous malformations or fistulae [9, 10]. However, cardiac magnetic resonance imaging (MRI), computed tomography angiography (CTA) chest, or ventilation-perfusion (VQ) studies, which could identify some of these other potential causes, were not pursued in this patient.

Coronary embolisms can manifest as a non-ST-segment elevation myocardial infarction (NSTEMI) like in our patient or an ST-segment elevation myocardial infarction (STEMI) as seen in other case reports (Table 1). The condition has also been seen in the setting of a concomitant stroke and diffuse systemic embolization [11]. The National Cerebral and Cardiovascular Center (NCVC) criteria for the clinical diagnosis of coronary artery embolism (CE) was proposed by Shibata et al. and uses combinations of major and minor criteria to categorize the diagnosis of CE into definite or probable [8] (Table 2). In that study, out of the 52 patients, 32 met the definitive criteria and 20 the probable criteria. Based on the proposed criteria, our patient would fall under the category of “probable” CE given the angiographic findings and risk factor of atrial fibrillation (one major and one minor criterion). Although no embolic source was found on echocardiography, the absence does not rule out CE.

In our case report, the patient was managed medically with anticoagulation given his high risk for coronary intervention, but additional therapeutic options exist that are consistent with other causes of ACS including stent placement, intracoronary thrombolysis, and thrombus aspiration—which is generally favored because of the decreased risk of distal embolization [1].

## 4. Conclusion

Coronary embolism is an unusual and therefore likely an underreported etiology of ACS. Patients often have varying levels of concurrent atherosclerosis which can further confound the picture. Distinctions can, however, be made angiographically. Management is the same for both: anticoagulation, thrombolysis, and percutaneous coronary intervention (PCI). Thrombus aspiration may be considered in patients with a heavy thrombus burden. However, following initial management, it is vital to further investigate the source of emboli in these patients. It is important to contemplate atrial fibrillation not only as a consequence of ACS but also as a cause of it. Additionally, it is important to consider bleeding risks given the need for both anticoagulation and antiplatelet therapy in these groups of patients.

## Figures and Tables

**Table 1:** Reported cases of coronary artery embolism with embolus location and outcomes.

Author	Patient age	Patient gender	History of atrial fibrillation or flutter	Peak troponin	Embolus location	Outcomes	References
Antoine et al.	58	M	Yes.	Troponin I 9.56 (unknown units).	Distal left circumflex artery (LCX).	NSTEMI managed by embolectomy.	[12]

Camaro et al.	66	F	Yes, new-onset.	Unknown.	Distal right coronary artery (RCA).	STEMI managed conservatively with anticoagulation after failed PCI.	[13]

Diaz et al.	52	M	Yes, new-onset.	Troponin I 0.6 ng/mL (normal < 0.024).	Proximal left anterior descending artery (LAD).	NSTEMI managed by thrombectomy.	[14]

Everett et al.	30	M	No.	Unknown.	Distal LAD.	Acute coronary syndrome managed by thrombectomy and anticoagulation.	[9]

Garg et al.	53	F	New-onset atrial fibrillation.	Troponin T 5.0 ng/mL (normal < 0.10 ng/mL).	Posterolateral branch of the LCX.	NSTEMI managed with anticoagulation.	[15]

Kotooka et al.	88	F	Yes.	Unknown.	Proximal RCA.	STEMI managed by thrombectomy.	[16]
	50	M	No.	Unknown.	Ostium of RCA.	STEMI treated by thrombus aspiration.	
	85	M	Yes.	Unknown.	Left main coronary artery (LCA).	STEMI complicated by cardiogenic shock, managed by dobutamine and an intra-aortic balloon pump and subsequently a thrombectomy and stent placement.	

Koutsampasopoulos et al.	69	F	Yes.	High sensitivity troponin T 5.33 ng/L (N < 0.014).	Midsegment of LAD.	STEMI managed with thrombectomy.	[17]

Nakano et al.	82	F	Yes.	Troponin I 0.65 ng/mL.	Unclear. ECG showed atrial fibrillation, an inverted T wave in V1-V3, an abnormal Q wave in V1 -V4, and ST-segment elevation in V3 and V4.	The patient was too unstable for an angiogram, thus was treated with intravenous heparin but died within 24 hours (diagnosis suspected by criteria proposed by Shibata et al.).	[11]

OSullivan et al.	70	M	Perioperative atrial fibrillation after noncardiac surgery with subsequent embolization.	High sensitivity troponin T 0.540 *μ*g/L (normal < 0.014).	Complete occlusion of RCA up to ostium.	STEMI managed with thrombectomy.	[18]

Sakai et al.	72	M	Yes.	Unknown, CKMB peaked at 2929 IU/L (normal < 180).	Proximal RCA.	STEMI managed by thrombectomy.	[19]

Takenaka et al.	65	F	Yes.	Unknown.	Distal LAD.	STEMI managed by balloon angioplasty and thrombolysis.	[20]

Van de Walle et al.	64	M	Yes.	Unknown.	Midsegment of LAD.	STEMI managed by stenting.	[1]

Zakaria et al.	65	F	New-onset atrial flutter.	Unknown.	Origin of LCX.	NSTEMI managed by thrombectomy.	[21]

**Table 2:** Proposed National Cerebral and Cardiovascular Center (NCVC) criteria for the clinical diagnosis of coronary artery embolism (CE) [1].

Major criteria
Angiographic evidence of CE and thrombosis without atherosclerotic components
Concomitant coronary artery embolization at multiple sites^a^
Concomitant systemic embolization without left ventricular thrombus attributable to acute myocardial infarction
Minor criteria
<25% stenosis on coronary angiography, except for the culprit lesion
Evidence of an embolic source based on transthoracic echocardiography transesophageal echocardiography, computed tomography, or magnetic resonance imaging
Presence of embolic risk factors: atrial fibrillation, cardiomyopathy, rheumatic valve disease, prosthetic heart valve, patent foramen ovale, atrial septal defect, history of cardiac surgery, infective endocarditis, or hypercoagulable state
Definite CE
Two or more major criteria, or
One major criterion plus ≥ 2 minor criterion, or
Three minor criteria
Probable CE
One major criterion plus 1 minor criterion, or
Two minor criteria
A diagnosis of CE should not be made if there is
Pathological evidence of atherosclerotic thrombus
History of coronary revascularization
Coronary artery ectasia
Plaque disruption or erosion detected by intravascular ultrasound or optic coherence tomography in the proximal part of the culprit lesion

The present proposed diagnostic criteria for CE include three major and three minor criteria. Weighted scoring of the criteria is used to differentiate between definite and probable CE in patients with acute myocardial infarction. ^a^Multiple vessel within one coronary artery territory or multiple vessels in the coronary tree. Note: this table is reproduced from Nakano H, Yamagami H, Ofuchi H. A case report of systemic embolic events associated with atrial fibrillation. Acute Medicine & Surgery 2017;4:12730.doi:10.1002/ams2.235.

## Supplementary Materials

Supplementary Materials Left heart catheterization showing three focal transluminal, translucent areas in the ostial, mid, and distal circumflex artery that was mobile and hazy appearing—concerning for emboli.
